# Progress Toward Rubella and Congenital Rubella Syndrome Elimination — Worldwide, 2012–2022

**DOI:** 10.15585/mmwr.mm7308a2

**Published:** 2024-02-29

**Authors:** Alan C. Ou, Laura A. Zimmerman, James P. Alexander, Natasha S. Crowcroft, Patrick M. O’Connor, Jennifer K. Knapp

**Affiliations:** ^1^Global Immunization Division, Global Health Center, CDC; ^2^Department of Immunization, Vaccines, and Biologicals, World Health Organization, Geneva, Switzerland.

**Summary**

**What is already known about this topic?**

Rubella virus infection during early pregnancy can result in miscarriage, fetal death, or live births with a myriad of physical defects (congenital rubella syndrome). Countries can eliminate rubella.

**What is added by this report?**

During 2012–2022, the percentage of World Health Organization countries including rubella vaccines in their vaccination schedule increased from 68% to 90%, and the percentage of the world’s infants vaccinated against rubella increased from 40% to 68%. From 2013 to 2021, global rubella incidence declined 81%. Enhanced surveillance for congenital rubella syndrome has resulted in increased detection of cases.

**What are the implications for public health practice?**

Substantial global progress toward rubella elimination has accelerated since 2012; however, the universal introduction of the rubella vaccine is essential in all countries to accelerate progress toward elimination.

## Abstract

Rubella virus is a leading cause of vaccine-preventable birth defects. Infection during pregnancy can result in miscarriage, fetal death, stillbirth, or a constellation of birth defects, including cataracts, deafness, heart defects, and developmental delay, known as congenital rubella syndrome (CRS). A single dose of rubella-containing vaccine can provide lifelong protection against rubella. The Global Vaccine Action Plan 2011–2020 included a target to achieve elimination of rubella in at least five of the six World Health Organization (WHO) regions by 2020, and rubella elimination is a critical goal of the Immunization Agenda 2030. This report updates a previous report and describes progress toward rubella and CRS elimination during 2012–2022. During 2012–2022, among 194 WHO countries, the number that included rubella-containing vaccine (RCV) in their immunization schedules increased from 132 (68%) to 175 (90%) and the percentage of the world’s infants vaccinated against rubella increased from 40% to 68%. Reported rubella cases declined 81%, from 93,816 in 2012 to 17,407 in 2022. Verification of rubella elimination was achieved in 98 (51%) of 194 countries by 2022, an increase from 84 (43%) countries in 2019. Despite significant progress in the introduction of RCV into routine immunization programs worldwide, approximately 25 million infants annually still do not have access to RCV. Nevertheless, even in complex settings, the increasing number of countries that have achieved and sustained rubella elimination demonstrates progress toward global rubella elimination.

## Introduction

Rubella virus is a leading cause of vaccine-preventable birth defects and can cause epidemics. Rubella virus infection usually produces a mild febrile rash illness in children and adults. However, infection during pregnancy, especially during the first trimester, can result in miscarriage, fetal death, stillbirth, or a constellation of birth defects known as congenital rubella syndrome (CRS). A single dose of rubella-containing vaccine (RCV) can provide lifelong protection against rubella (*1*). The Global Vaccine Action Plan 2011–2020 (GVAP) included a target to achieve elimination of rubella in at least five of the six World Health Organization (WHO) regions by 2020 (*2*), and WHO recommended capitalizing on the accelerated measles elimination activities as an opportunity to introduce RCV (*1*). In 2020, the Measles and Rubella Strategic Framework (MRSF) 2021–2030 (*3*) was developed under the Immunization Agenda 2030 (IA2030) (*4*), which includes rubella elimination as a critical impact goal. MRSF includes guidance at the country, regional, and global levels for planning and implementing more effective measles and rubella elimination efforts. This report updates a previous report (*5*) and summarizes global progress toward elimination of rubella and CRS from 2012 (when accelerated rubella control activities were initiated) through 2022.

## Methods

### Immunization Activities

The WHO-recommended strategy for introducing RCV into national immunization programs is through an initial catch-up vaccination campaign targeting persons who might not have been naturally exposed to rubella (usually children and adolescents aged ≤14 years) (*1*). WHO recommends that countries then achieve and maintain at least 80% coverage with ≥1 dose of RCV delivered through routine services or campaigns (*1*).

Each year, countries report immunization data to WHO and UNICEF using the electronic Joint Reporting Form (eJRF).* This form includes information on immunization schedules and the number of vaccine doses administered through routine immunization services and vaccination campaigns. WHO and UNICEF estimate coverage with the first and second RCV doses delivered through routine immunization services^†^ for all countries, using annual administrative coverage data, national coverage estimates, and vaccination coverage surveys. For this report, 2012–2022 eJRF data were analyzed, with a focus on data from 2012 (the new phase of rubella vaccine introduction and elimination), 2020 (the beginning of the COVID-19 pandemic), and 2022 (the most recent data available). Because RCV first became available in high-income countries, World Bank income groupings for 2022 were used to evaluate income-related disparities in RCV introduction and coverage at the national level.^§^

### Surveillance Activities and Reported Rubella and CRS Incidence

Rubella and CRS surveillance data are reported through eJRF using standard case definitions (*5*). Rubella surveillance relies on the measles surveillance system to detect cases of febrile rash illness. CRS cases are detected through separate surveillance systems, often using a few sentinel sites that might not be nationally representative (*6*). The Global Measles and Rubella Laboratory Network comprises 743 laboratories that conduct measles and rubella case confirmation through serologic and molecular testing. For this report, rubella and CRS surveillance data were reviewed, including the distribution of rubella virus genotypes. This activity was reviewed by CDC, deemed not research, and was conducted consistent with applicable federal law and CDC policy.^¶^

### Monitoring of Progress Toward Elimination

Progress toward regional goals is measured by the number of countries introducing RCV and the number verified as having eliminated rubella and CRS. The interruption of endemic rubella virus transmission is defined as no ongoing local rubella transmission for ≥12 months. When interruption of transmission is sustained for 36 months, an independent regional verification commission verifies countries as having eliminated rubella (*7*). Data on verification of elimination are available in the Regional Verification Commission reports.**^,^^††^^,^^§§^^,^^¶¶^^,^***

## Results

### Immunization Activities

**FIGURE 1:**
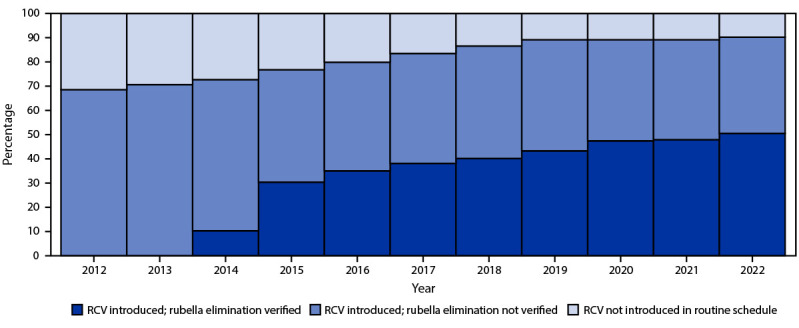
Percentage of World Health Organization countries that introduced rubella-containing vaccine into the routine immunization schedule and the percentage with verified rubella elimination, by year (N = 194) — worldwide, 2012–2022 **Abbreviation:** RCV = rubella-containing vaccine.

In 2022, RCV had been introduced in 175 (90%) of 194 countries,^†††^ a 33% increase compared with the 132 (68%) countries that offered RCV in 2012 (Figure 1). All countries in the Region of the Americas (AMR), the European Region (EUR), the South-East Asia Region (SEAR), and the Western Pacific Region (WPR) have introduced RCV. In the two remaining regions, RCV has been introduced in 32 (68%) of 47 countries in the African Region (AFR) and 17 (81%) of 21 countries in the Eastern Mediterranean Region (EMR) (Table).

**TABLE:** Progress toward control and elimination of rubella and congenital rubella syndrome, by World Health Organization region — worldwide, 2012, 2019, and 2022

Characteristic	WHO region (no. of countries)						
	AFR (47)	AMR (35)	EMR (21)	EUR (53)	SEAR (11)	WPR (27)	Worldwide (194)
**Regional rubella or CRS target**	Elimination in 80% of countries	Regional elimination	None	Regional elimination	Regional elimination	Regional elimination	**None**
Countries verified eliminated, no. (%)*							
2012	NA	NA	NA	NA	NA	NA	**NA**
2019	NA	35 (100)	NA	45 (91)	NA	4 (15)	**84 (43)**
2022	NA	35 (100)	4 (19)	50 (94)	4 (36)	5 (19)	**98 (51)**
**Countries with RCV in schedule, no. (%)**							
2012	3 (6)	35 (100)	14 (67)	53 (100)	5 (45)	22 (81)	**132 (68)**
2019	31 (66)	35 (100)	16 (76)	53 (100)	11 (100)	27 (100)	**173 (89)**
2022	32 (68)	35 (100)	17 (81)	53 (100)	11 (100)	27 (100)	**175 (90)**
**Regional rubella vaccination coverage, %^†^**							
2012	0	94	36	95	5	86	**40**
2019	32	87	43	96	93	94	**69**
2022	36	84	42	93	92	92	**68**
**Countries reporting rubella cases, no. (%)**							
2012	38 (81)	34 (97)	18 (86)	45 (85)	11 (100)	20 (74)	**166 (86)**
2019	42 (89)	33 (94)	18 (86)	47 (89)	10 (91)	19 (70)	**169 (87)**
2022	41 (87)	27 (77)	16 (76)	41 (77)	11 (100)	13 (48)	**149 (77)**
**Reported rubella cases, no.**							
2012	10,751	15	1,490	30,535	6,877	44,148	**93,816**
2019	5,981	25	2,322	627	4,537	35,067	**48,559**
2022	10,021	0	2,678	29	3,728	951	**17,407**
**Countries reporting CRS cases, no. (%)**							
2012	18 (38)	34 (97)	9 (43)	41 (77)	6 (55)	15 (56)	**123 (63)**
2019	16 (34)	31 (89)	12(57)	40 (75)	7 (64)	17 (63)	**123 (63)**
2022	20 (43)	31 (89)	16 (76)	42 (79)	10 (91)	14 (52)	**133 (69)**
**Reported CRS cases, no.**							
2012	69	3	20	62	14	133	**301**
2019	9	0	21	8	358	22	**418**
2022	5	0	933	2	554	33	**1,527**

**Abbreviations:** AFR = African Region; AMR = Region of the Americas; CRS = congenital rubella syndrome; EMR = Eastern Mediterranean Region; EUR = European Region; NA = not available; RCV = rubella-containing vaccine; SEAR = South-East Asia Region; WHO = World Health Organization; WPR = Western Pacific Region.

* Established regional verification commissions verify achievement of elimination in five regions (AMR, EMR, EUR, SEAR, and WPR).  ^† ^RCV coverage estimates are determined by WHO and UNICEF Estimates of National Immunization Coverage.

**FIGURE 2:**
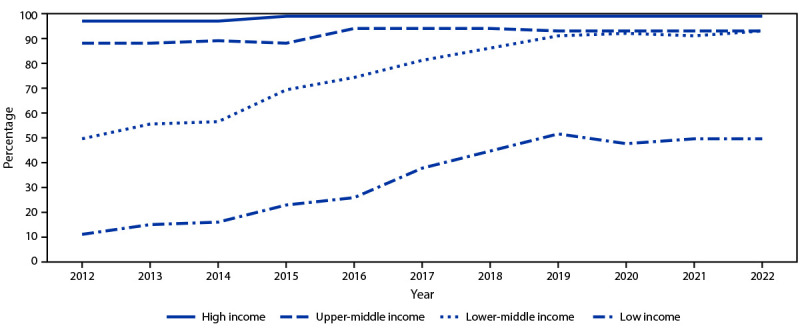
Percentage of World Health Organization countries that have introduced rubella-containing vaccine into the routine immunization schedule, by historical World Bank income group* and year (N = 194) — worldwide, 2012–2022^†^ * Gross national income per capita in U.S. dollars for 2022: high income ≥ $13,846; upper-middle income = $4,466–$13,845; lower-middle income = $1,136–$4,465; and low income ≤$1,135. https://datahelpdesk.worldbank.org/knowledgebase/articles/906519-world-bank-country-and-lending-groups ^†^ In 2022, there were 59 high-income, 49 upper-middle–income, 51 lower-middle–income, 13 low-income, and 22 unclassified countries.

The introduction of RCV within low- and lower-middle–income countries has increased steadily over time (Figure 2). In 2012, RCV had been introduced in only 11% of 36 low-income countries and 50% of 46 lower-middle–income countries; however, by 2022, RCV introduction had increased to include 13 (50%) of 26 low-income countries and 51 (94%) of 54 lower-middle–income countries.

According to the WHO/UNICEF Estimates of National Immunization Coverage, coverage with the first dose of RCV globally among infants increased from 40% in 2012 to 68% in 2022, with wide regional variation (range = 36% [AFR]–93% [EUR]) (Table). In 2022, rubella vaccination coverage was 27% in low-income countries, 70% in lower-middle–income countries, 88% in upper-middle–income countries, and 93% in high-income countries. Excluding those countries that have not yet introduced RCV, 2022 coverage was 82% in low-income countries, 81% in lower-middle–income countries, 86% in upper-middle–income countries, and 94% in high-income countries.

### Surveillance Activities and Reported Rubella and CRS Incidence

The number of countries reporting rubella cases, including the reporting of zero cases, increased from 166 (86%) in 2012 to 169 (87%) in 2019. During the COVID-19 pandemic, the number of countries reporting cases declined to 144 (74%) in 2020, and then increased slightly to 149 (77%) in 2022, but overall, remained below 2012 levels (Table). The number of countries reporting CRS cases remained constant at 123 (63%) in 2012 and 2019 but increased to 133 (69%) in 2022.

Compared with the 93,816 rubella cases reported in 2012, reported rubella cases declined 48%, to 48,559 in 2019, and decreased further to 17,407 in 2022 (Table). Reported CRS cases increased from 301 in 2012 to 418 in 2019 and 1,527 in 2022, primarily as the result of initiation of CRS surveillance and reporting in several populous countries (Afghanistan, Bangladesh, India, Indonesia, and Pakistan) since 2012.

During 2012–2022, a total of 5,722 rubella sequences from 45 countries were reported to the Rubella Virus Nucleotide Surveillance database in the Global Measles Rubella Laboratory Network. Among these, 3,295 (58%) were genotype 1E, and 2,395 (42%) were genotype 2B. However, 67% and 24% of the sequences were from China and Japan, respectively, highlighting the need to enhance global virologic surveillance for rubella (Min-hsin Chen, CDC, personal communication, 2024).

### Progress Toward Elimination

Five WHO regions now have rubella and CRS regional elimination goals; AFR established a goal in 2021 (*8*). Although EMR has yet to set an elimination goal, the region has committed to achieving elimination (*4*). The AMR commission verified that the entire region had eliminated rubella and CRS in 2015; verification commissions in AFR, EMR, EUR, SEAR, and WPR assess rubella elimination status on a country-by-country basis. The number of countries in which elimination of endemic rubella has been verified has increased from 84 in 2019 to 98 countries in 2022: none in AFR, 35 (100%) in AMR, four (19%) of 21 in EMR, 50 (94%) of 53 in EUR, four (36%) of 11 in SEAR, and five (19%) of 27 in WPR.

## Discussion 

Rubella elimination has accelerated since 2012; by 2022, elimination had been verified in 51% of the world’s countries. During 2019–2022, despite COVID-19 disruptions, 15 countries were verified as having achieved elimination. In addition, rubella elimination has been verified in nearly 25% of lower-middle–income countries, illustrating that rubella can be eliminated in complex socioeconomic circumstances. Furthermore, endemic transmission has not been reestablished in any country that has been verified to have achieved elimination, likely attributable to high vaccine efficacy and lifelong immunity conferred by a single dose of the vaccine and to sustained immunization coverage at levels necessary for herd immunity *(**1**)*. The increased commitment by countries, regions, and other international stakeholders to eliminate rubella has driven this considerable progress.

Since the onset of the intensified efforts to eliminate rubella in 2012, the number of countries that have introduced RCV and the coverage achieved have both increased substantially.^§§§^ From 2012 to 2022, the number of countries that have introduced RCV increased by approximately one third, from 132 to 175, and global RCV immunization coverage increased from 40% to 68%. The pace of vaccine introduction slowed during the COVID-19 pandemic, with only Comoros and Pakistan introducing RCV in 2021. Although the number of low-income and lower-middle–income countries introducing RCV has increased, and the overall number of reported rubella cases has declined 81% during 2012–2022, approximately 25 million infants annually still lack access to RCV, more than one half of them living in low-income, conflict-affected areas.

The increase in reported CRS cases since 2012 reflects an increase in the number of countries conducting CRS surveillance in 2022. Because surveillance for CRS is limited in scope within countries and many countries do not conduct CRS surveillance, the number reported in 2022 represents a vast underestimate of the actual number of CRS cases globally. Though modeled estimates of CRS demonstrated a two-thirds reduction in the global burden during 2010–2019, more than 32,000 infants are born with CRS each year, primarily in countries that have not introduced RCV (*9*). With intensified investments, countries that have introduced RCV are likely to eliminate rubella. However, the threat of reintroduction remains until every country has introduced the vaccine (*10*). Therefore, it is essential that every country introduces RCV to achieve global rubella elimination.

### Limitations

The findings in this report are subject to at least three limitations. First, ascertaining the accuracy and reliability of surveillance and immunization data remains a challenge, limiting the ability to identify immunity gaps, focus on immunization-strengthening activities, and demonstrate the interruption of rubella virus transmission. Second, during the COVID-19 pandemic, decreased reporting by countries and the quality of surveillance data reported limited the ability to monitor progress during the previous 3 years. Finally, because many countries do not conduct CRS surveillance and because sentinel surveillance only identifies those infants who have access to specialty hospitals for diagnosis and treatment, CRS surveillance is limited in its accuracy.

### Implications for Public Health Practice

The 19 countries that have yet to introduce rubella vaccine include approximately 25 million infants, mostly living in settings classified by the World Bank as conflict-affected and low-income.^¶¶¶^ To redress this equity gap, these countries need support in introducing rubella vaccine. Because of extensive disruptions to routine immunization programs during the COVID-19 pandemic, ensuring all children are up to date with rubella vaccination is essential, especially those who missed vaccination during the pandemic. Additional strategies to immunize adolescents and adults are needed to protect against rubella infection throughout the life course to ensure adults of childbearing age are protected from the risk of having an infant with CRS.

## Footnotes

* https://www.who.int/teams/immunization-vaccines-and-biologicals/immunization-analysis-and-insights/global-monitoring/who-unicef-joint-reporting-process

† Calculated for the first dose of RCV (RCV1), among children aged 1 year or, if RCV1 is given at age ≥1 year, among children aged 24 months. Calculated for the second dose of RCV (RCV2) among children at the recommended age for the administration of RCV2, per the national immunization schedule. https://immunizationdata.who.int/

§ World Bank publishes annual gross national income classification cutoffs per capita in U.S. dollars. The 2023 fiscal year provides classification data through 2022: high income ≥$13,846; upper-middle income = $4,466–$13,845; lower-middle income = $1,136–$4,465; and low income ≤$1,135. https://datahelpdesk.worldbank.org/knowledgebase/articles/906519-world-bank-country-and-lending-groups

¶ 45 C.F.R. part 46.102(l)(2), 21 C.F.R. part 56; 42 U.S.C. Sect. 241(d); 5 U.S.C. Sect. 552a; 44 U.S.C. Sect. 3501 et seq.

**
https://applications.emro.who.int/docs/WHOEMEPI362E-eng.pdf

††
https://www.who.int/europe/publications/i/item/WHO-EURO-2023-7719-47486-69809

§§
https://iris.who.int/handle/10665/370787

¶¶
https://iris.who.int/handle/10665/365590

***
https://www.paho.org/en/news/29-4-2015-americas-region-declared-worlds-first-eliminate-rubella

††† Among the 19 countries that have not yet introduced RCV, four are in EMR (Afghanistan, Djibouti, Somalia, and Sudan) and 15 are in AFR (Central African Republic, Chad, Democratic Republic of the Congo, Equatorial Guinea, Ethiopia, Gabon, Guinea, Guinea-Bissau, Liberia, Madagascar, Mali, Niger, Nigeria, South Africa, and South Sudan).

§§§
https://doi.org/10.1016/j.ijid.2023.10.012

¶¶¶
https://thedocs.worldbank.org/en/doc/608a53dd83f21ef6712b5dfef050b00b-0090082023/original/FCSListFY24-final.pdf

## References

[1] World Health Organization. Rubella vaccines: WHO position paper—July 2020. Geneva, Switzerland: World Health Organization; 2020. https://iris.who.int/bitstream/handle/10665/332952/WER9527-306-324-eng-fre.pdf?isAllowed=y&sequence=1

[2] World Health Organization. Global vaccine action plan 2011–2020. Geneva, Switzerland: World Health Organization; 2013. https://iris.who.int/handle/10665/78141

[3] World Health Organization. Measles and rubella strategic framework: 2021–2030. Geneva, Switzerland: World Health Organization; 2020. https://iris.who.int/bitstream/handle/10665/339801/9789240015616-eng.pdf?sequence=1&isAllowed=y

[4] World Health Organization. Immunization agenda 2030: a global strategy to leave no one behind. Geneva, Switzerland: World Health Organization; 2020. https://www.who.int/publications/m/item/immunization-agenda-2030-a-global-strategy-to-leave-no-one-behind

[5] Zimmerman LA, Knapp JK, Antoni S, Grant GB, Reef SE. Progress toward rubella and congenital rubella syndrome control and elimination—worldwide, 2012–2020. MMWR Morb Mortal Wkly Rep 2022;71:196–201. 10.15585/mmwr.mm7106a2 35143468 PMC8830626

[6] Patel MK, Gibson R, Cohen A, Dumolard L, Gacic-Dobo M. Global landscape of measles and rubella surveillance. Vaccine 2018;36:7385–92. 10.1016/j.vaccine.2018.10.007 30318167

[7] World Health Organization. Guidance for evaluating progress towards elimination of measles and rubella. Wkly Epidemiol Rec 2018;93:544–52. https://iris.who.int/bitstream/handle/10665/275394/WER9341-544-552.pdf?sequence=1&isAllowed=y

[8] World Health Organization Regional Office for Africa. Framework for the implementation of the Immunization Agenda 2030 in the WHO African Region. Brazzaville, Republic of the Congo: World Health Organization Regional Office for Africa; 2021. https://www.afro.who.int/sites/default/files/2021-07/AFR-RC71-7%20Framework%20for%20the%20implementation%20of%20the%20Immunization%20Agenda%202030%20in%20the%20WHO%20African%20Region.pdf

[9] Vynnycky E, Knapp JK, Papadopoulos T, Estimates of the global burden of congenital rubella syndrome, 1996–2019. Int J Infect Dis 2023;137:149–56. 10.1016/j.ijid.2023.09.003 37690575 PMC10689248

[10] Winter AK, Lambert B, Klein D, Feasibility of measles and rubella vaccination programmes for disease elimination: a modelling study. Lancet Glob Health 2022;10:e1412–22. 10.1016/S2214-109X(22)00335-7 36113527 PMC9557212

